# Cytogenetic assessment of Iranian infertile men with undescended testis: A retrospective study

**DOI:** 10.5935/1518-0557.20200006

**Published:** 2020

**Authors:** Neda Sharifi, Marjan Sabbaghian, Faramarz Farrahi, Navid Almadani, Parnaz Borjian Boroujeni, Anahita Mohseni Meybodi

**Affiliations:** 1 Department of Molecular Genetic, Faculty of Basic Sciences and Advanced Technologies in Biology, University of Science and Culture, Tehran, Iran; 2 Reproductive Biomedicine Research Center, Royan Institute for Reproductive Biomedicine, Department of Genetics, ACECR, Tehran, Iran; 3 Reproductive Biomedicine Research Center, Royan Institute for Reproductive Biomedicine, Department of Andrology, ACECR, Tehran, Iran

**Keywords:** cytogenetics, undescended testis, cryptorchidism, male infertility, Iranian population

## Abstract

**Objective:**

Undescended testis (UDT) is a urogenital disease that affects fertility. This study looked into the cytogenetic abnormalities of Iranian infertile patients with UDT.

**Methods:**

Our study included 522 infertile patients with UDT (case group) and two control groups, one with 300 infertile men without UDT and another with 268 fertile men.

**Results:**

Chromosomal abnormalities were found in 45 patients with UDT (8.62%). Seven of the alterations were considered as normal features. Klinefelter syndrome and mosaicism were the most common anomalies. Chromosomal abnormalities were found in 31 infertile men in the control group (10.33%), 13 of which deemed normal and 18 (6%) anomalous. Nine chromosomal abnormalities were found in the second control group with fertile men (3.35%), six deemed normal and three (1.11%) anomalous.

**Conclusion:**

Despite the high rate of abnormalities in infertile controls (6%) and the higher rate seen in infertile individuals with UDT indicate a significant prevalence of chromosomal abnormalities in the Iranian population, particularly when the literature suggests that the normal rate of abnormal karyotypes should be within the 0.7-1% range in the general population. The incidence of abnormal karyotypes increased when infertile patients had additional conditions such as UDT.

## INTRODUCTION

Undescended testis (UDT) or cryptorchidism is one of the most prevalent urogenital defects in boys that lead to sexual development deficiency. Testicles usually descend during the last weeks of gestation or a few weeks after birth (Ghirri *et al*., 2002). The process unfolds in two phases, in which the testicles descend from the intra-abdominal location into the extra-abdominal scrotal sac of the boy (Kaleva & Toppari, 2005). The first phase is trans-abdominal and androgen-independent, while the second is androgen-dependent. The first phase occurs from the 8^th^ to the 15^th^ week and the second from the 25^th^ to the 35^th^ week of gestation. The reasons for UDT are still unknown. UDT has been linked to a number of hormone disorders and related factors (Cox *et al*., 2008), including testosterone, gonadotropin-releasing hormone (GnRH), follicle stimulating hormone (*FSH*), luteinizing hormone (*LH*), anti-Müllerian hormone (*AMH*), insulin-like 3 protein (*INSL3*), and *HOXA 10* (Niedzielski *et al*., 2016).

The prevalence of UDT situates between 2-4% in full-term boys (Komarowska *et al*., 2015). Two possible consequences of UDT are testicular cancer and infertility (Giwercman *et al*., 1987; Deng *et al*., 2019). UDT can be unilateral or bilateral. The incidence of infertility is significantly higher in patients with bilateral UDT than in subjects with unilateral UDT (Hollowell, 2014). According to previous studies, subjects with unilateral UDT are usually more successful at having children than their counterparts with bilateral UDT (Cendron *et al*., 1989). Genetic disorders account for 15-30% of all cases of male infertility (Neto *et al*., 2016). Genetic screening may benefit patients with azoospermia or oligospermia (Cavkaytar *et al*., 2012). Karyotyping as a first step in genetic investigation helps doctors to determine whether their patients present with chromosomal aberrations. Chromosomal abnormalities can be generally categorized as numerical or structural.

Numerical abnormalities include cases in which the patient is missing one or more chromosomes or has one or a few extra chromosomes. Structural aberrations include duplications, deletions, inversions, translocations, insertions, rings, and isochromosomes (Genetic Alliance & District of Columbia Department of Health, 2010). Since chromosomal disorders are commonly seen with infertility, we assumed that the incidence of chromosomal abnormalities might be high in infertile patients with UDT. This study was the first to examine the cytogenetic alterations of infertile patients with UDT to understand the association between UDT and karyotype abnormalities in the Iranian population.

## MATERIALS AND METHODS

### Patients and clinical data

This retrospective study included 522 infertile men with undescended testis (UDT) in the case group and two control groups, the first with 268 fertile men who underwent sex selection for family balancing at the Royan Institute and had at least one child, and the second with 300 infertile men without UDT or urogenital disease who sought fertility treatment at the Royan Institute. Our study included individuals seen at the Royan Institute from 2010 to 2015. All participants gave written consent before joining the study. The Ethics Committee of the Royan Institute approved the study. The individuals included in this study had previously undergone physical examination, hormone testing, semen analysis, and karyotyping. The subjects in the fertile control group had normal spermograms, FSH, LH, and testosterone levels. A specialist performed the physical examination of patients with UDT and reported the type of UDT and additional information about the appearance of sex organs with the aid of ultrasound examination. The patients with UDT did not have other urogenital diseases such as hypospadias or ambiguous genitalia. Subjects with UDT were further divided into bilateral and unilateral UDT. The hormone tests of infertile patients were performed by electrochemiluminescence (ECL) and included FSH, LH, and testosterone. Semen analysis was performed after 2-5 days of sexual abstinence at the andrology laboratory according to the World Health Organization (WHO) criteria (WHO, 2010). Sperm concentration, semen volume, pH, motility, and morphology were thoroughly checked. Infertile patients were categorized as having oligospermia/severe oligospermia (sperm counts of less than 5 million per ml) or azoospermia (zero sperm count). Statistical analysis was performed on SPSS version 22. Differences between groups with a *p*-value ≤0.05 in the chi-square test were deemed significant.

### Cytogenetic analysis

Karyotyping was performed on trypsin-banded metaphase chromosomes with a standard protocol of 550 band resolutions from peripheral lymphocyte cultures. Then, 50 random metaphase spreads were analyzed for each person. More than 50 metaphase spreads were checked in patients suspected for mosaicism. Karyotypes were defined using the International System for Human Cytogenetic Nomenclature (ISCN 2016). Chromosome variations such as centromeric heterochromatin variants were considered as normal variations based on the ISCN 2016 (McGowan-Jordan *et al*., 2016) and previous studies (Zhou *et al*., 2006). Although previous studies have correlated inv (9) (p12q13) with male infertility due to spermatogenesis disorders, this finding was considered a normal feature in our study in accordance with the ISCN (Sasiadek *et al*., 1997; Mozdarani *et al*., 2007).

### Statistical analysis

The Chi-square test was used in statistical analysis. A *p*-value ≤0.05 was considered significant.

## RESULTS

Our study included Iranian men divided as follows: 522 infertile patients with UDT; 300 infertile men without UDT; and 268 fertile men. Participant ages ranged from 25 to 61 years at the time of diagnosis; participants were aged 40±5.6 years on average. The included patients belonged to different ethnic groups. Physical examination, ultrasound, or patient medical records indicated that 292 individuals had unilateral UDT (55.94%) and 230 subjects had bilateral UDT (44.06%). Semen analysis of patients with UDT showed that 348 (66.66%) were azoospermic, 110 (21.07%) were oligospermic, and 64 (12.26%) had severe oligospermia. The group of infertile individuals had 204 (68%) subjects with azoospermia, 70 (23.33%) with oligospermia, and 26 (8.66%) with severe oligospermia. The hormone profile of patients with UDT revealed increased gonadotropin levels and lower to nearly normal plasma testosterone levels.

Mean FSH and LH levels were 26.89±22.91 and 11.89±9.13 mIU/mL, respectively, while the mean testosterone level was 3.47±2.44 ng/mL. The hormone profiles of infertile individuals showed mean FSH and LH levels of 13.15±12.84 and 10.21±9.88 mIU/mL, respectively, and an average testosterone level of 3.61±2.13 ng/mL. The ranges for normal hormone levels based on WHO criteria were as follows: FSH (1.5-12) mIU/mL; LH (1-10) mIU/mL; testosterone (2-8) ng/mL. Cytogenetic analysis revealed chromosomal alterations in all three groups, with 45 individuals (8.62%) in the case group, nine (3.35%) in the fertile control group, and 31 (10.33%) in the infertile group without UDT. Normal chromosomal alterations were seen in seven individuals (1.34%) in the case group, six cases (2.23%) in the fertile group, and 13 (4.33%) subjects in the infertile group.

Normal variations were excluded from statistical analysis. Thirty-eight subjects with UDT (7.3%), three (1.11%) fertile individuals, and 18 (6%) infertile patients had different kinds of pathogenic chromosomal abnormalities. Table 1 describes in detail the chromosomal alterations seen in the case and control groups. Numerical chromosomal abnormalities in the case group featured Klinefelter syndrome and chromosomal mosaicism in 18 (3.44%) and 10 cases (1.91%), respectively. Interestingly, Klinefelter syndrome was the most common chromosomal anomaly. One patient with UDT had 47, XYY syndrome. Nine of the patients with mosaic karyotypes had sex chromosome mosaicism; one had mosaicism of unknown origin (probably linked to the Y chromosome); and one patient had structural and numerical chromosomal abnormalities (Table 1).

**Table 1:** Chromosomal alterations observed in study participants

	Chromosomal Alterations*							
	Normal Variations		Chromosomal Abnormalities					
			Numerical		Structural		Combined numerical and structural	
	Type	No of Cases	Type	No of Cases	Type	No of Cases	Type	No of Cases
Infertile Patients with UDT N=522	46,XY, inv (9)(p12q13) 46,XY, inv(3)(p11q11.2) 46,X, inv(Y)(p11.2q11.2)	4 2 1	Klinefelter Syndrome Jacobs Syndrome Different types of Klinefelter Syndrome mosaicism 47,XX,+mar[4]/46,XX[12] 45,X[ ]/46,XY[]	18 1 4 1 4	46,XY,t(1;16)(p10;q10) 46,XY,t(13;16)(q12.1;q22) 45,XY,der(13;13)(q10;q10) 46,XY,inv(4)(p15.32p14) 46,X,del(Y)(q11.222)	1 1 1 1 1	45,X[21]/46,X,dic(Y;Y)? (p11.32; p11.2)[13]	1
Fertile Men (1^st^ Control Group) N=268	46,XY,inv(9)(p12q13) 46,XYqh+ 46,XY,18qh+ 46,XY,21pstk+ 46,XY,15ps+	2 1 1 1 1	47,XXY,[1]/45,X,[1]/46,XY[13] 48,XXYY[1]/45,X[1]/46,XY[13]	11	46,XY,inv(10)(p12.3q21.3)	1	-	-
Infertile Patients without UDT (2^ed^ control group) N=300	46,XY,inv(9)(p12q13) 46,XY,1qh+ 46,XY,9qh+ 46,XY,16qh+ 46,XY,14pss,14stk+ 46,XY,15pss,15pstk+ 46,XY,15pstk+	7 1 1 1 1 1 1	Klinefelter Syndrome mosaicism 47,XXY,[2]/46,XY[13] 45,X[21]/46,XY[18]	13 1 1	46,XY,dup(7)(q11,21q11.22) 46,XY,t(6;12)(q25.1;q21.31) 46,XY,t (6;17)(p21,33;p10)	1 1 1	-	-

* Four cases of unfertile patients with UDT diagnosed with sex reversal 46, XX syndrome are not mentioned in this table.

**Figure 1:**
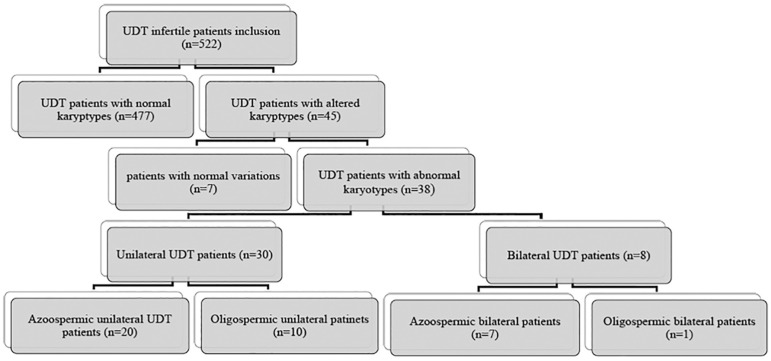
Categorization of patients with UDT based on clinical features.

The five structural abnormalities observed were Robertsonian and reciprocal translocations, deletion of the (Y) chromosome, and an inversion. Another small group of abnormal karyotypes comprised four cases of sex reversal (0.76%) with the 46, XX karyotype instead of the 46, XY normal karyotype, which features were described in a published study developed at the Royan Institute (Mohammadpour Lashkari *et al*., 2017). The analysis of abnormal karyotypes in the infertile control group revealed that 13 (4.33%) individuals had Klinefelter syndrome, a number as high as the one observed in the case group. The group also featured two individuals with mosaicism, two with translocations, and one with duplication. Thirty patients in the case group had unilateral UDT and eight had bilateral UDT. The incidence of abnormal karyotypes was higher among individuals with unilateral UDT. Hormone profiles showed that patients with UDT and abnormal karyotypes had higher mean FSH and LH levels (27.98±19.11 and 17.52±13.7 mIU/mL, respectively) and lower to nearly normal testosterone levels (2.64±1.69 ng/mL). Semen analysis of the subjects in the case group with abnormal karyotypes revealed 27 cases of azoospermia and 11 of oligospermia. The incidence of azoospermia was higher than the incidence of oligospermia in patients with UDT (Figure 1). The statistical analysis of chromosomal alterations revealed a significant difference (*p*-value ≤0.05) between case and control groups.

## DISCUSSION

Although UDT is a multifactorial condition, the genetic factors linked to the condition are still a topic of discussion. This retrospective study looked into cytogenetic alterations in Iranian infertile patients with UDT and compared them against fertile and infertile controls. The novelty in this study lies in the fact that it is the largest cytogenetic study ever performed in an Iranian population. Our study found chromosomal abnormalities in 7.3% of the infertile individuals with undescended testicles. The rates of chromosomal aberrations in infertile controls without UDT and fertile controls were 6% and 1.11%, respectively. Statistical analysis revealed a significant difference between the case and control groups (*p*-value ≤0.05). An earlier study analyzed 110 patients with UDT and/or hypospadias. The authors reported seven abnormal karyotypes among patients (6.4%) (Yamaguchi *et al*., 1991). Another study found seven abnormal karyotypes (4.4%) in a group of 160 patients with UDT (Sasagawa *et al*., 1996). Karyotyping of an 11-month-old boy with bilateral undescended testes revealed he had 47, XYY syndrome (Suzuki *et al*., 1999), as seen in one of our patients. A total of 984 individuals with hypospadias and/or UDT were studied in 2002; 884 of them had UDT and a reported rate of chromosomal anomalies of 1.84%.

In agreement with previous studies, the authors recommended that patients with UDT should undergo chromosomal analysis, but mentioned testing was more beneficial for patients with combined congenital abnormalities since they were at greater risk of having higher rates of abnormalities (Moreno-Garcia & Miranda, 2002). We also recommended karyotyping for individuals with UDT. Interestingly, although our study enrolled fewer individuals, the prevalence of abnormal karyotypes was significantly higher in our population (7.3%). The differences between the two populations - our study enrolled 522 infertile individuals with UDT only - may explain this apparent discrepancy. Another study included 48 individuals with UDT and hypospadias. Eight (16.7%) had abnormal karyotypes. Surprisingly, despite the high frequency of chromosomal abnormalities in their patients, the authors reported that karyotyping was not needed for all individuals with hypospadias or UDT (McAleer & Kaplan, 2001). Cox *et al*. (2008) reported that karyotyping is not required in patients with only unilateral or bilateral UDT or in patients with UDT and distal hypospadias. The authors recommended karyotyping only for patients with proximal hypospadias and UDT, since they accounted for a greater proportion of chromosomal abnormalities (16%). The observations described by McAleer & Kaplan (2001) and Cox *et al*. (2008) did not match our findings, since our patients with UDT only had a high incidence of abnormal karyotypes (7.3%). This number appears to indicate a significant prevalence of chromosomal abnormalities in the Iranian population when compared with 6% and 1.11% found in infertile and fertile controls, respectively, and even more so when the literature suggests that the normal rate of abnormal karyotypes should be within the 0.7-1% range in the general population (Nussbaum *et al.*, 2016).

In a group of 94 boys with Klinefelter syndrome, 83.7% had the 47, XXY karyotype and 7.1% had 47, XXY/ 46, XY mosaicism. UDT was the most diagnosed disease among prepubertal individuals with Klinefelter syndrome. The phenotypes diagnosed in pubertal patients included small testes, UDT, and gynecomastia (Pacenza *et al*., 2012). Our study also found an association between UDT and Klinefelter syndrome, a combination seen in 18 cases (3.44%) of infertile individuals in our population. Klinefelter syndrome was likewise observed in 13 infertile individuals without UDT (4.33%). Other types of sex chromosome mosaicism were seen in 1.91% of the individuals in the case group, a higher proportion than in the general population. Our results showed that other chromosomal abnormalities such as sex reversal and structural aberrations may be related to UDT, although with lower incidence compared with Klinefelter syndrome. The rate of chromosomal abnormalities in our study was high among infertile controls without UDT, with an incidence of 6% *vs.* 7.3% in the case group. It seems, however, that patients with more significant involvement - such as UDT - are more likely to have chromosomal abnormalities. The comparison of hormone profiles of patients with UDT and individuals in the case group with UDT and abnormal karyotypes revealed that all subjects with UDT had increased FSH and LH levels and lower to nearly normal testosterone levels. These features may be a consequence of UDT, not a trait resulting from having abnormal karyotypes.

## CONCLUSION

In agreement with previous studies, our study found a significant association between UDT and karyotype abnormalities, although the proportion of individuals with karyotype abnormalities was higher in the Iranian population included in our study (7.3%) than the proportion seen in the general population. Karyotyping should be offered to patients with UDT undergoing fertility treatment, since they may have chromosomal abnormalities and infertility caused by chromosomal anomalies. Preimplantation genetic diagnosis (PGD) is strongly recommended for azoospermic patients tested positive for chromosomal abnormalities in microsurgical testicular sperm extraction (Micro-TESE) and individuals with oligospermia.
